# Microsatellite-Based Genetic Diversity and Population Structure of Huacaya Alpacas (*Vicugna pacos*) in Southern Peru

**DOI:** 10.3390/ani13091552

**Published:** 2023-05-05

**Authors:** Deyanira Figueroa, Flor-Anita Corredor, Ruben H. Mamani-Cato, Roberto F. Gallegos-Acero, Nicoll Condori-Rojas, Richard Estrada, Lizeth Heredia, Wilian Salazar, Carlos Quilcate, Carlos I. Arbizu

**Affiliations:** 1 Dirección de Desarrollo Tecnológico Agrario, Instituto Nacional de Innovación Agraria (INIA), Av. La Molina 1981, Lima 15024, Peru; 2 Dirección de Desarrollo Tecnológico Agrario, Instituto Nacional de Innovación Agraria (INIA), Estación Experimental Agraria Illpa, Puno 21002, Peru; 3 Facultad de Medicina Veterinaria y Zootecnia, Universidad Nacional del Altiplano, Av. Floral 1153, Puno 21001, Peru; 4 Facultad de Ingeniería y Ciencias Agrarias, Universidad Nacional Toribio Rodríguez de Mendoza de Amazonas (UNTRM), Cl. Higos Urco 342, Chachapoyas 01001, Peru

**Keywords:** SSR, camelids, Andean region, genetics

## Abstract

**Simple Summary:**

This research employs fourteen and twelve microsatellite markers for two Huacaya alpaca populations from Southern Peru to estimate the genetic diversity and population structure. Our findings show that these two populations (Quimsachata and Ajoyani) were not structured. We also found that both populations have a large amount of diversity, which is a good indicator for conservation measures. These findings provide useful indicators regarding conservation programs created for this species to ensure their sustainable use.

**Abstract:**

The alpaca population mostly consists of the Huacaya phenotype and is widely distributed in Southern Peru. This study aimed to estimate the genetic diversity and population structure of two Huacaya alpaca populations (Ajoyani and Quimsachata) using fourteen and twelve microsatellite markers for each population, respectively. A total of 168 alpaca biological samples were outsourced to Peruvian laboratories for DNA extraction and genotyping. For genetic diversity, observed heterozygosity (H_o_), expected heterozygosity (H_e_), polymorphism information content (PIC), and fixation indices values were estimated. An admixture analysis was performed for the population structure analysis. Different programs were used for these estimations. In total, 133 (Ajoyani) and 129 (Quimsachata) alleles were found, with a range of 4 to 17 by locus. The mean H_O_, H_E_, and PIC per marker for Ajoyani were 0.764 ± 0.112, 0.771 ± 0.1, and 0.736; for Quimsachata, they were 0.783 ± 0.087, 0.773 ± 0.095, and 0.738, respectively. The population structure showed no structure with K = 2. This study provides useful indicators for the creation of appropriate alpaca conservation programs.

## 1. Introduction

In Peru, the alpaca is a livestock species adapted to the high altitudes of the Andes. More than 3.6 million alpacas, or more than 80% of the world’s entire population, live in Peru. The highlands of Peru, including Puno, Cusco, and Huancavelica, are home to the majority of the world’s alpacas [1]. Alpacas are phenotypically separated into two subpopulations according to their type of fleece: Huacaya and Suri, with Huacaya accounting for more than 80% of the total population and Suri accounting for the remaining 20% [2]. This animal is resilient to cold temperatures and survives with little human management. Therefore, it has survival advantages over introduced livestock, such as sheep and cattle, in the Andes.

Alpaca farming is an activity of great importance for a population that lives at more than 4000 m above sea level and which has the highest rates of poverty [3,4]. The alpaca is an important source of income for many rural families in Peru. These animals are raised primarily for their wool, which is used in the production of high-quality garments in international markets [5]. They also provide meat and manure to be used as fertilizer in the local markets [6]. The breeding of alpacas in Peru takes advantage of extensive areas of natural grasslands, which due to factors associated with altitude, could not be used efficiently by other domestic animals [7,8].

Molecular markers have proven to be powerful tools to assess genetic variation and relationships in animal species, presenting various identification methods [9]. Microsatellites, which are short nucleotide sequences that vary from one individual to another, make it possible to identify the degree of polymorphisms and the level of repetitiveness contained in the entire genome, which favors the individual identification of each member in a population under study [10].

There are some previous studies focused on the genetic diversity and population structures of various south American camelids, such as alpacas, llamas, guanacos, and vicuñas [11,12,13,14,15,16,17], mainly in the regions of Peru and Bolivia. In addition, other members of the Camelidae family have been studied [18,19,20,21]. In the vast majority, low-density microsatellite markers or mitochondrial markers were used. Barreta et al. [11] and Echalar et al. [13] both studied Huacaya alpacas from Bolivia and found high levels of genetic diversity. Barreta et al. [11] studied the genetic diversity and population structure in eight different locations in Bolivia. Using 22 microsatellites, they found 58 alleles and identified between 4 and 18 alleles per locus with high levels of observed heterozygosity ranging from 0.61 to 0.69. Echalar et al. [13] studied the intraspecific genetic analysis of Bolivian alpacas and the interspecific relationship between llamas and vicuñas. In general, the populations of alpacas and llamas present greater genetic diversity (H_e_ = 0.73) than vicuñas (H_e_ = 0.59). The authors concluded that there are low genetic differentiation values among the alpaca populations studied, and high levels of genetic diversity among three species (alpacas, llamas, and vicuñas). In the case of Peru, the studies of Morón et al. [14], Vallejo et al. [9], and Yalta et al. [22] used between 10 and 15 microsatellite markers to infer genetic diversity in Huacaya alpacas. Vallejo et al. [9], in a study for the southern region of Peru, identified 213 alleles and detected 12 to 25 alleles per locus, finding high levels of H_o_ with a range of 0.79 to 0.90. Similarly, Morón et al. [14] found 225 alleles and detected 15 loci, finding high levels of genetic diversity (H_e_ = 0.826) in three farms in the central zone of Peru.

These results show that the use of microsatellite markers has been useful and informative to infer the genetic diversity of Huacaya alpaca populations. Despite being the world’s largest breeder of alpacas, there are relatively few studies that describe the alpaca populations in Peru. Studies of genetic relationships provide useful information from within-population patterns of genetic variation at marker loci and allow the deduction of demographic factors important for diversity conservation [10,23]. Therefore, this study will use microsatellites to assess the genetic diversity and population structure of two Huacaya alpaca populations: A Camelid Germplasm Bank alpaca population whose biodiversity represents Peru’s patrimony and a breeders’ association in Peru’s most alpaca-populated region. The objective of this study is to assess genetic variation among two populations of Huacaya alpaca from Southern Peru, an area with a high concentration of these animals that is under the effects of climate change.

## 2. Materials and Methods

### 2.1. Sample Collection

**Figure 1:**
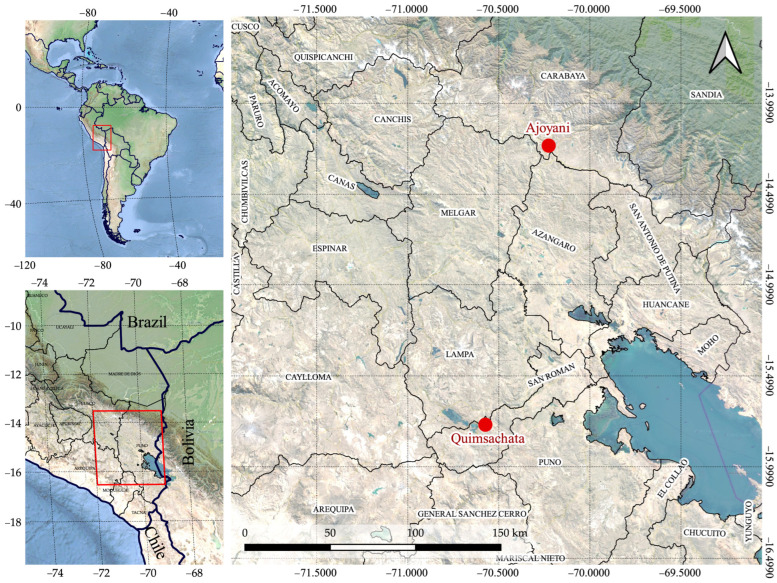
Geographical localization of Quimsachata and Ajoyani alpaca populations in the department of Puno, Southern Peru.

The sample collection from alpaca specimens was conducted in accordance with Peruvian National Law No. 30407: “Animal Protection and Welfare”. Two Huacaya alpaca populations reared in the department of Puno, Southern Peru, were used in this study. Camelid Germplasm Bank population (Quimsachata for the rest of the text), located in the district of Santa Lucia, Province of Lampa, and ‘‘AURORA APROCSA AJOYANI’’ population (Ajoyani for the rest of the text) located in the district of Ajoyani, Province of Carabaya (Figure 1). Quimsachata and Ajoyani alpaca populations are located in different agroecological zones, which are classified into dry and humid puna, respectively.

Biological material for a total of 168 nonrelated animals was collected for genotyping. We gained access to blood and follicle samples. From the Quimsachata population, 116 Huacaya alpacas were analyzed from samples of leukocytes extracted from whole blood obtained by jugular vein puncture, and 52 Huacaya alpacas from the Ajoyani population from three different breeders were analyzed from samples of hair follicles. Individuals were coded by correlative numbers prior to analysis.

### 2.2. Genomic DNA Extraction, and Microsatellite Genotyping

Quimsachata samples were outsourced to Bionoma (Lima, Peru) to conduct the genomic DNA extraction and microsatellite genotyping, and for the determination of microsatellite alleles, twelve microsatellite loci described for alpacas and llamas (LCA5, LCA66, LCA71, LCA77, LCA94, LCA99, YWLL36, YWLL29, YWLL40, YWLL44, LCA19, and LCA8) [24,25,26,27] were amplified in two multiplex PCR reactions using a Qiagen Multiplex PCR Kit (Qiagen) in a model 9700 GeneAmp^®^ thermal cycler (Applied Biosystems, Foster City, CA, USA). The amplified products were separated using capillary electrophoresis, using the ABI3137 XL genetic analyzer (Applied Biosystems). For the analysis of the samples, the 400HD ROX size standard was used. Allele size assignment in base pairs (bp) was carried out using 446 alpaca DNA samples from six different places to make a bin set and their naming was carried out using the Genemapper v4 program.

Ajoyani samples were outsourced to Instituto Nacional de Innovación Agraria (Lima, Peru) to conduct the genomic DNA extraction and microsatellite genotyping, and for the determination of microsatellite alleles, fourteen microsatellite loci recommended for the International Society for Animal Genetics (ISAG) (LCA5, LCA8, LCA19, LCA24, LCA37, LCA56, LCA65, LCA66, LCA94, LCA99, LGU49, YWLL29, YWLL40, and YWLL46) were amplified through PCR reactions using a Mastercycler Pro S (Eppendorf Inc., Hamburg, Germany). The amplified products were separated using capillary electrophoresis, using the ABI3130 XL genetic analyzer (Applied Biosystems). For the analysis of the samples, the LIZ600 size standard was used. Allele size assignment in base pairs (bp) was adjusted to the ISAG nomenclature and their naming was carried out using the Genemapper v4 program.

### 2.3. Genetic Diversity

We calculated the genetic diversity parameters such as the number of alleles (k), observed heterozygosity (H_o_), expected heterozygosity (H_e_), and the polymorphic information content (PIC) for each of the 14 microsatellites used for Ajoyani alpaca and each of the 12 microsatellite markers for the Quimsachata alpaca, using Cervus v3.0.7 software [28]; the fixation index among individuals within a population (F_is_) was estimated using Arlequin v3.5.2. software [29]. The Hardy–Weinberg equilibrium test was performed for each locus using Cervus software [28].

### 2.4. Population Structure

The population genetic structure assessment was performed using STRUCTURE v2.3.4 software [30], with a burn-in period set to 50,000 and 500,000 Markov chain Monte Carlo (MCMC) iterations. A total of 15 independent runs were performed (K = 1 to K = 15), and these were repeated 10 times to check the consistency of the results. STRUCTURE HARVESTER v0.7 program [31] was used to select the best K in all of the following stages and was visualized using the CLUMPAK program [32].

## 3. Results

A total of 133 different alleles were found across fourteen microsatellite loci in the Ajoyani population (Table 1) and 129 were found across twelve microsatellite loci in the Quimsachata population (Table 2). All of the microsatellites are polymorphic since all have more than one allele. The number of alleles ranged from four (YWLL46) to fourteen (LCA66) in the Ajoyani population and from five (LCA71) to fifteen (YWLL44) in the Quimsachata population. The mean observed heterozygosity overall loci in the Ajoyani population was 0.764 + −0.112, while expected heterozygosity was 0.771 + −0.1. The mean observed heterozygosity overall loci in the Quimsachata population was 0.783 + −0.087, while expected heterozygosity was 0.773 + −0.095. The mean PIC was 0.736 and 0.738 for Ajoyani and Quimsachata, respectively (Table 3).

F_is_ value was calculated in Arlequin; eight makers had negative F_is_ estimates (LCA5, LCA94, YWLL29, YWLL40, LCA19, LCA8, LCA24, and LGU49) in the Ajoyani population (Table 4) and seven markers (LCA5, LCA71, LCA94, LCA99, YWLL4, LCA19, and LCA8) had negative F_is_ estimates in the Quimsachata population (Table 5). The average F_is_ for all loci was 0.009 for the Ajoyani population and −0.017 for the Quimsachata population. The study found significant deviations from the Hardy–Weinberg equilibrium in one locus in the Ajoyani population (LCA66) and, similarly, for one in the Quimsachata population (LCA71), suggesting nonrandom pairings in the populations of Huacaya alpaca.

**Figure 2:**
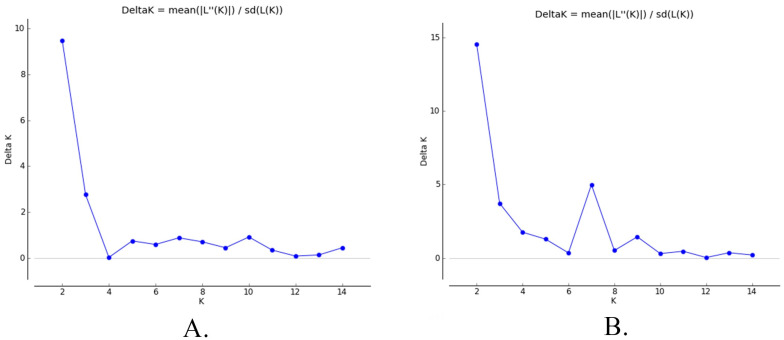
Delta K values for the STRUCTURE HARVESTER analysis for Ajoyani (**A**) and Quimsachata (**B**) populations.

In addition, from the same data, the analysis of the population structure was carried out based on the admixture level of each alpaca individual using the STRUCTURE program [30]. K = 2 (Figure 2) is the most optimal value for both the Ajoyani and Quimsachata populations

**Figure 3:**
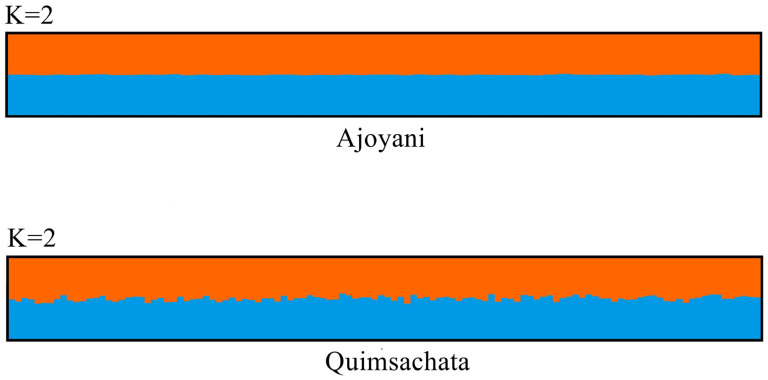
Graphical representation of the clustering outcomes suggested K = 2 for the Ajoyani and Quimsachata populations. The proportion of each color indicates the probability of each individual being divided into the corresponding cluster.

Visualization with CLUMPAK showed no structure. Each alpaca was represented by a single vertical line broken into colored segments (Figure 3).

## 4. Discussion

In this study, we explored the genetic diversity and relationships of two populations of alpacas from different altitudes and agroecological zones in the highlands of the Southern Andean Cordillera in Peru. Quimsachata is a germplasm bank of in vivo and ex situ colored alpacas, whose objective is to maintain the greatest diversity of alpacas of the Huacaya and Suri phenotypes and the greatest diversity of fleece colors; Quimsachata does not sell male or female alpacas for reproduction. Ajoyani has, as its main objective, the breeding of alpacas of the Huacaya phenotype and for the white fleece, whose selection objective is the quantity and quality of the fiber; for this purpose, they resort to the purchase of breeders from the district from Macusani (a district near Ajoyani).

Overall, the Quimsachata and Ajoyani alpaca populations showed high levels of diversity. All of the microsatellites were considered useful for diversity studies because they have four or more alleles per loci [33]. The least polymorphic loci were four (YWLL46) and five (LCA71) alleles in Ajoyani and Quimsachata, respectively (Table 1). In a study by Barreta et al. [17], who analyzed over 22 microsatellites in Bolivian alpacas, four alleles were also determined in the least polymorphic marker.

The average number of alleles per locus in the Ajoyani and Quimsachata populations was 9.5 and 10.75, respectively (Table 2). Similarly, La Manna et al. [34] determined the average number of alleles (9.67) in 65 samples. La Manna et al. [34] and our results are less than the average number of alleles for alpacas from Puno (13.4) [22], three regions in Peru (Huancavelica, Junin, and Puno) (14.40) [35], and Bolivian alpacas (11.73) [11]; this may be as a result of the smaller sample size compared to the other studies (183, 265, and 149, respectively). In all previous studies, Quimsachata possessed similar or even higher alleles than Ajoyani, showing greater genetic diversity.

Most of the markers are highly polymorphic (PIC > 0.5) [36], except for YWLL46 (PIC = 0.453) in the Ajoyani population and LCA71 (PIC = 0.457) in the Quimsachata population, which means that the microsatellite markers used in this study are reliable and shows a high genetic diversity of the Huacaya alpaca population. The Quimsachata population’s average PIC (0.738) was slightly higher than the Ajoyani’s (0.736), but it was lower than Puno’s (0.8301) [22], the three Peruvian areas’ (Huancavelica, Junin, and Puno) (0.798) [35], and Bolivian alpacas’ (0.826) [11]. The differences in PIC results from our study in comparison to the Bolivian and Puno studies could be due to smaller sample sizes in the Quimsachata and Ajoyani populations. In the Bolivian study [11], they examined a greater number of microsatellites, where they collected samples from eight different locations, and 22 microsatellites were analyzed. Furthermore, in the case of the Puno study [22], the animals sampled came from two breeding centers. The animals in the Puno research are from the open breeding nucleus, which influences their low levels of inbreeding depression and high migration rate compared to the Quimsachata population, resulting in a difference in PIC values for microsatellites.

Six loci in the Ajoyani population (LCA66, LCA99, LCA37, LCA56, LCA65, and YWLL46) and five loci in the Quimsachata population (LCA66, LCA77, YWLL36, YWLL29, and YWLL40) showed lower H_o_ than H_e_, less than the half of the total loci, which may indicate that there is neither an excess nor a deficiency of heterozygosity in the populations under study. This result is consistent with previous studies carried out by other authors [34,37,38].

The results showed that the Quimsachata population has high genetic diversity and a high number of alleles, and the average H_o_ and H_e_ are higher than the Ajoyani population, meaning that a well-managed process is being conducted or can be only attributed to the higher number of samples employed in this work. The Hardy–Weinberg equilibrium analysis showed that the frequencies of the markers evaluated in the two populations of Huacaya alpacas have remained stable among generations, which is consistent with the average F_is_ value. Overall, the studied loci were 0.009 in the Ajoyani population and −0.017 in Quimsachata; these values are not different from zero, suggesting no heterozygote deficit or excess. The structure analysis suggested two as the number of populations and the graphic shows admixture in all individuals, which means that there is no structure in Ajoyani and Quimsachata populations, but the Quimsachata population shows different levels of admixture between individuals, while Ajoyani seems more homogeneous. This can be explained by the different breeding objectives for each population.

## 5. Conclusions

In summary, Ajoyani and Quimsachata are two Huacaya alpaca populations from the highest alpaca-populated region of Peru. The genetic diversity of these two populations was determined using fourteen and twelve microsatellite markers that showed a high level of polymorphism. Because of the large number of alleles, the heterozygosity, and the high PIC values, both populations exhibited high genetic diversity. Quimsachata must have high genetic diversity as a germplasm bank, as evidenced by the average number of alleles (10.75) and average H_o_ (0.783 ± 0.087) and H_e_ (0.773 ± 0.095), but this could also be due to a higher number of Quimsachata individuals being analyzed. Furthermore, the structural analysis revealed that the populations have no structure. However, structure graphics showed that the Quimsachata population is slightly more heterogeneous than the Ajoyani. Finally, selected markers showed that they are very useful in genetic diversity studies.

## Figures and Tables

**Table 1:** Genetic diversity parameters for the Ajoyani population.

Microsatellite	Ajoyani			
	k	H_o_	H_e_	PIC
LCA5	8	0.804	0.756	0.71
LCA66	14	0.673	0.826	0.802
LCA94	7	0.808	0.785	0.748
LCA99	11	0.769	0.834	0.804
YWLL29	7	0.788	0.745	0.711
YWLL40	6	0.635	0.626	0.564
LCA19	11	0.788	0.744	0.708
LCA8	11	0.904	0.845	0.819
LCA24	7	0.75	0.702	0.654
LCA37	13	0.827	0.849	0.824
LCA56	11	0.827	0.855	0.83
LCA65	12	0.8	0.877	0.854
LGU49	11	0.885	0.844	0.818
YWLL46	4	0.442	0.508	0.453
TOTAL	133	-	-	-

k: number of alleles at the locus, H_o_: observed heterozygosity, H_e_: expected heterozygosity, and PIC: polymorphic information content.

**Table 2:** Genetic diversity parameters for the Quimsachata population.

Microsatellite	Quimsachata			
	k	Ho	He	PIC
LCA5	10	0.802	0.755	0.713
LCA66	10	0.733	0.744	0.696
LCA71	5	0.595	0.528	0.457
LCA77	17	0.828	0.859	0.843
LCA94	8	0.81	0.8	0.766
LCA99	13	0.862	0.832	0.808
YWLL36	13	0.853	0.868	0.849
YWLL29	9	0.759	0.77	0.739
YWLL40	6	0.664	0.694	0.636
YWLL44	15	0.888	0.871	0.853
LCA19	12	0.724	0.703	0.673
LCA8	11	0.879	0.848	0.825
TOTAL	129	-	-	-

k: number of alleles at the locus, H_o_: observed heterozygosity, H_e_: expected heterozygosity, and PIC: polymorphic information content.

**Table 3:** Average genetic diversity parameters for each population.

Population	k	H_o_	H_e_	PIC
Ajoyani	9.5	0.764 ± 0.112	0.771 ± 0.1	0.736
Quimsachata	10.75	0.783 ± 0.087	0.773 ± 0.095	0.738

k: number of alleles at the locus, H_o_: observed heterozygosity, H_e_: expected heterozygosity, and PIC: polymorphic information content.

**Table 4:** Fixation index among individuals within the population (F_is_) and deviation from Hardy–Weinberg (HWD) for the microsatellites studied in the Ajoyani population.

Microsatellite	Ajoyani	
	F_is_	HWD
LCA5	−0.064	NS
LCA66	0.187	**
LCA94	−0.029	NS
LCA99	0.078	NS
YWLL29	−0.06	NS
YWLL40	−0.014	NS
LCA19	−0.06	NS
LCA8	−0.07	NS
LCA24	−0.07	NS
LCA37	0.026	NS
LCA56	0.033	NS
LCA65	0.089	NS
LGU49	−0.048	NS
YWLL46	0.131	NS

*p*-value (NS = not significant, ** *p* < 0.01).

**Table 5:** Fixation index among individuals within the population (Fis) and deviation from Hardy–Weinberg (HWD) for the microsatellites studied in the Quimsachata population.

Microsatellite	Quimsachata	
	F_is_	HWD
LCA5	−0.062	NS
LCA66	0.015	NS
LCA71	−0.127	*
LCA77	0.037	NS
LCA94	−0.012	NS
LCA99	−0.036	NS
YWLL36	0.017	NS
YWLL29	0.015	NS
YWLL40	0.043	NS
YWLL44	−0.02	NS
LCA19	−0.031	NS
LCA8	−0.038	NS

*p*-value (NS = not significant, * *p* < 0.1).

## Data Availability

The data presented in this study are openly available in Dryad at https://doi.org/10.5061/dryad.mkkwh7153 (accessed on 8 March 2023).
